# Fixed Drug Eruption Associated with Sulfonamides Sold in Latino Grocery Stores — Greater Washington, DC, Area, 2012–2013

**Published:** 2013-11-22

**Authors:** Christine C. Yang, Audrey N. Green, Scott A. Norton

**Affiliations:** State University of New York Upstate Medical University; Mercer Univ School of Medicine; Children’s National Medical Center

In March 2012, a Salvadoran-American boy aged 7 years living in Maryland developed three slightly painful, well-demarcated, flat, gray-brown patches on his torso. A dermatologist in Washington, DC, suspected a fixed drug eruption (an erythema multiforme-like adverse drug reaction that occurs in the same location each time the person uses a particular medication). The child had recently taken a cough and cold remedy, Baczol Antigripal, which was made in El Salvador and purchased in a Maryland suburb of Washington, DC, without a prescription. The Baczol Antigripal ingredients included the sulfonamide-containing antibiotic trimethoprim-sulfamethoxazole (TMP/SMX), which is a common cause of fixed drug eruption. In June 2013, another Salvadoran-American child, a girl aged 14 years living in northern Virginia, was evaluated for a similar fixed drug eruption likely caused by a Baczol product purchased near her home. In August 2013, staff members from the Children’s National Medical Center investigated the availability of Baczol products in grocery stores in Salvadoran neighborhoods of Washington, DC, and neighboring suburbs. TMP/SMX-containing products were found in seven of 19 stores.

Four Baczol products were identified; two listed TMP/SMX as an ingredient and were labeled for sale in El Salvador only. TMP/SMX is a known cause of fixed drug eruptions and other adverse drug reactions and cannot be legally dispensed in the United States without a prescription. On August 20, the Food and Drug Administration (FDA) issued a Safety Alert in English and Spanish.* The FDA advised purchasers of these products to stop taking it and consult a health-care professional. Clinicians should be aware that patients might consume nonprescribed antibiotics and should specifically ask about over-the-counter cold and flu remedies, especially when an adverse drug reaction is suspected.

The dermatologist who diagnosed the first case and learned of the sale of Baczol Antigripal in Maryland notified Maryland’s Department of Health and Mental Hygiene that an antibiotic was being sold without a prescription. In April 2012, the department issued an alert regarding Baczol, which is sold in El Salvador as an over-the-counter remedy for the common cold and cough in children. The FDA has issued import refusals for those Baczol formulations that contain TMP/SMX (1). Use of nonprescribed TMP/SMX poses a public health risk because of possible adverse drug reactions (2) and spread of antibiotic resistance; however, the availability of such drugs is unknown. An investigation was conducted to determine the availability of Baczol products in grocery stores in Salvadoran neighborhoods of Washington, DC, and neighboring suburbs.

Two investigators for the Children’s National Medical Center used U.S. Census data to identify heavily Salvadoran neighborhoods in the Washington, DC, area and then searched the Internet for Latino grocery stores in Washington, DC (Columbia Heights and Adams Morgan neighborhoods), Virginia (Falls Church, Alexandria, and Springfield), and Maryland (Wheaton and Silver Spring). Search terms used included “Latino market,” “Latino grocery,” “Latin American market,” and “Latin American grocery.” The investigators identified areas within each neighborhood that appeared to have several Latino grocery stores in close proximity. They visited identified stores and conducted scripted questioning for Baczol. Latino grocery stores nearby that had not been found via the Internet search also were assessed with the same scripted materials. The investigators also searched the medical literature but found no cases of fixed drug eruption attributed to sulfonamide-containing drugs sold without prescription.

TMP/SMX-containing products were found in three of seven stores in Washington, DC, one of six stores in Virginia, and three of six stores in Maryland. Two products, Baczol Antigripal (Figure) and Baczol Expectorante, listed TMP/SMX as an ingredient. The labels for both products, written entirely in Spanish, stated that they were for sale in El Salvador only (Table). The label for Baczol Expectorante stated explicitly that it was for sale without a prescription. A third TMP/SMX-containing product identified by investigators was Bactrizole, which is made by a different manufacturer in El Salvador (Table). None of the three TMP/SMX-containing medications described potential adverse drug reactions on their packaging.

Two additional products, also called Baczol Antigripal (Figure) and Baczol Expectorante, did not contain TMP/SMX. The labels for these products, written in Spanish and English, but otherwise nearly identical to those of their sulfonamide-containing counterparts, stated that they were for sale in the United States without a prescription and had appropriate National Drug Code numbers.

## Editorial Note

A survey in South Carolina showed that 20% of Latino immigrants have obtained or purchased oral antibiotics without a prescription in the United States (3). In most cases, the purchaser sought to treat common illnesses, often caused by viruses, including the common cold, ear infections, cough, sore throat, and diarrhea.

Self-medication with antibiotics for viral illnesses is not consistent with prudent use of antibiotics. TMP/SMX is a known cause of fixed drug eruptions and other rare but potentially serious adverse drug reactions, such as Stevens-Johnson syndrome, toxic epidermal necrolysis, and bone marrow suppression (4). Compared with other antibiotic classes, sulfonamides, including the sulfamethoxazole that is contained in TMP/SMX, are responsible for higher rates of moderate-to-severe allergic reactions, hospitalizations, and hematologic or renal effects (5).

What is already known on this topic?

Self-medication with oral antibiotics obtained without a prescription has been observed among substantial numbers of persons in U.S. Latino communities.

What is added by this report?

Following reports of severe skin conditions in youths, approximately one third of Latino grocery stores surveyed in the greater Washington, DC, area were found to be selling illegally imported sulfonamide antibiotic preparations.

What are the implications for public health practice?

Clinicians should be aware that patients with severe skin conditions might have consumed nonprescribed antibiotics and should specifically ask about over-the-counter cold and flu remedies, especially when there is a suspicion of an adverse drug reaction. Health-care professionals and consumers are encouraged to report adverse events related to Baczol Antigripal and Baczol Expectorante to the Food and Drug Administration’s MedWatch Safety Information and Adverse Event Reporting Program.

One Baczol formulation also contained metamizole (or dipyrone), a nonsteroidal antiinflammatory drug formerly widely prescribed outside the United States (under trade names including Analgin, Dipirona, Novalgin, and Optalgin) as an analgesic and antipyretic, and banned by the FDA in 1977 because of its association with agranulocytosis (6). The drug is still commonly used in developing countries as an analgesic and antipyretic. One study reported that 25% of surveyed Latinos who have used metamizole in the United States purchased it within the United States (7). Although this study did not address the availability of metamizole in Latino grocery stores, it was found in a Baczol Antigripal formulation.

This investigation showed that medications containing TMP/SMX are readily available from many Latino grocery stores in Washington, DC, and its suburbs. These products, Baczol Antigripal and Baczol Expectorante, might be confused with similar products legally sold in the United States. The legal formulations have the same names and nearly identical packaging as their TMP/SMX-containing counterparts. In response to this investigation, the FDA issued a Safety Alert in English and Spanish on August 20, 2013, advising consumers who have purchased one of these products to immediately stop taking it and consult a health care professional. Clinicians should be aware that patients might consume nonprescribed antibiotics and should specifically ask about over-the-counter cold and flu remedies, especially when there is a suspicion of an adverse drug reaction. Health-care professionals and consumers are encouraged to report any adverse events related to Baczol Antigripal and Baczol Expectorante to FDA’s MedWatch Safety Information and Adverse Event Reporting Program.†

## Figures and Tables

**FIGURE:**
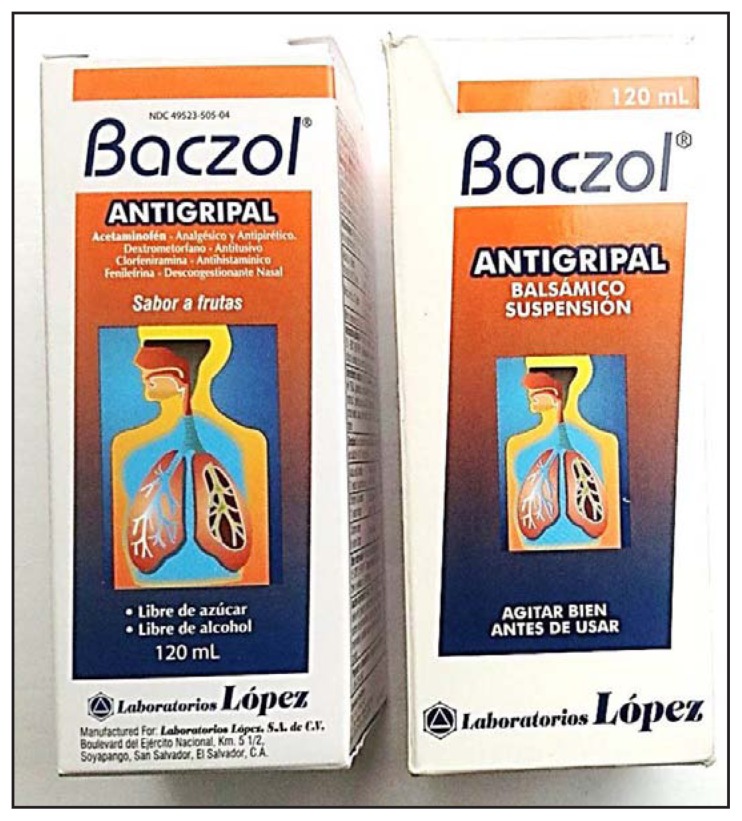
Two Baczol products purchased without a prescription from Latino grocery stores in the greater Washington, DC, area: (left) a product with no sulfonamide component, which was exported legally to the United States; (right) a product with TMP-SMX, which according to the label, is intended for sale solely in El Salvador and requires a prescription — 2013

**TABLE:** Properties of over-the-counter medications containing trimethoprim/sulfamethoxazole sold in Latino grocery stores — greater Washington, DC, area, 2012–2013

Product name	Indications for use	Recommended dose	Active ingredients (per 5-mL dose)	Contraindications	Label states product for sale solely in El Salvador	Prescription required in El Salvador
Baczol Antigripal	Influenza, pharyngitis, bronchopulmonary problems, fever, and general malaise	Adults and children aged ≥12 yrs: 20 mL Children aged 6–11 yrs: 10 mL Children aged 2–5 yrs: 5 mL Children aged <2 yrs: consult a physician Every 12 hrs	Trimethoprim 40 mg Sulfamethoxazole 200 mg Guaifenesin 50 mg Chlorpheniramine maleate 1 mg Phenylephrine HCL 2.5 mg Metamizole [dipyrone] 250 mg	Contraindicated for hypersensitivities to the components of the formula. Avoid use in pregnant or breastfeeding mothers.	Yes	Yes: “Venta bajo prescription médica”
Baczol Expectorante	Respiratory infections such as acute or chronic bronchitis or bronchial complications of viral diseases and bronchopneumonia, laryngitis, laryngotracheo-bronchitis	Adults: 20 mL Children aged 6–12 yrs: 5–10 mL Children aged 2–5 yrs: 5 mL Children aged 1–2 yrs: 2.5 mL Every 12 hrs	Trimethoprim 40 mg Sulfamethoxazole 200 mg Ambroxol HCl 15 mg	Contraindicated for hypersensitivities to the components of the formula. Avoid use in pregnant or breastfeeding mothers.	Yes	No: “Venta sin receta”
Bactrizole Balsámico Forte Suspensión	Indicated for bronchopulmonary infections resistant to antibiotics. Is a broad spectrum antibiotic agent effective in respiratory infections. Alleviates nasal obstruction.	Adults and children aged >12 yrs: 2 teaspoons (10 mL) every 6–12 hrs for 5–14 days	Trimethoprim 40 mg Sulfamethoxazole 200 mg Guaifenesin 50 mg Chlorpheniramine maleate 1 mg Phenylephrine HCL 2.5 mg	Hypersensitivity to any components. Pregnancy, lactation.	No	Yes: “Venta bajo receta médica.”

## Footnotes

* Available at http://www.fda.gov/drugs/drugsafety/ucm365650.htm.

† Available at http://www.fda.gov/medwatch/report.htm.
